# A Statement of Circumstances Connected with the Apothecaries' Act, and Its Administration

**Published:** 1817-06

**Authors:** 


*A Statement of Circumstances connected with the Apothecaries' Act, and its Administration.* By GEORGE MAN BURROWS, M.D. F.L.S. &c. Octavo, pp. 47, *with Appendix.* London, 1817.

THIS pamphlet having been circulated with the last number of the *London Medical Repository,* and thereby having obtained an extensive circulation among general practitioners, it is needless for us to take any measures for detailing its contents. But we may be allowed to say, that we have seldom had our feelings wound up to a higher degree of indignation than in the perusal of this interesting document. The indefatigable zeal, the unremitting perseverance, the unbounded enthusiasm, with which Dr. Burrows has, for many years, advocated the cause of a numerous, useful, and respectable class of medical society, are too well known to require comment here. But who will not blush for human nature, when he finds the generous exertions of such a man crossed and cavilled at, while he himself is vilified, traduced, and shamefully treated by a calculating, superannuated, narrow-minded junto of *Medico-Merchants,* whose most exalted ideas cannot soar beyond the *profits and loss* on an invoice of "*drugs, oils, and colours!*" When the dignity of the profession is entrusted to such hands, then, indeed, "farewell physic!" Dr. Burrows has given an able and lucid exposé of the whole affair, and that in terms at once gentleman-like, candid, and independent.

Valeat quantum valere debet!

---

PART

